# Supplementary material for: Epichaperome Inhibition by PU-H71-Mediated Targeting of HSP90 Sensitizes Glioblastoma Cells to Alkylator-Induced DNA Damage
Source: Cancers (Basel). 2024 Nov 24;16(23):3934. doi: 10.3390/cancers16233934 (PMC11640441; doi:10.3390/cancers16233934)

Supplementary Table 1 List of antibodies with their used dilution and manufacturer catalogue information

**Antibody**	**Manufacture**	**Catalogue information**	**Dilution**
HSP90	Cell Signaling Technology	CST#4877	1:1,000
HSP70	Cell Signaling Technology	CST#4872	1:1,000
Cleaved PARP	Cell Signaling Technology,	CST#5625	1:1,000
GAPDH	Santa Cruz Biotechnology, Dallas, Texas	sc-47724	1:1,000
p-EGFR (Tyr1068)	Cell Signaling Technology	CST#3777	1:1,000
EGFR	Santacruz Biotech	sc-373746	1:500
p-AKT (Ser 473)	Cell Signaling Technology	CST#4060	1:1,000
AKT	Cell Signaling Technology	CST#2920	1:1,000
pMAPK	Cell Signaling Technology	CST#4370	1:1,000
MAPK	Cell Signaling Technology	CST#4696	1:1,000
p-S6 (Ser 240/244)	Cell Signaling Technology	CST#2215	1:1,000
S6	Cell Signaling Technology	CST#2217	1:1,000
Anti-mouse IgG (H+L) (DyLight™ 680 Conjugate)	Cell Signaling Technology	CST#5470	1:15,000
Anti-rabbit IgG (H+L) (DyLight™ 800 4X PEG Conjugate)	Cell Signaling Technology	CST#5151	1:30,000

Original blots

Epichaperome Inhibition by PU-H71–Mediated Targeting of HSP90 Sensitizes Glioblastoma Cells to Alkylator-Induced DNA Damage

Pratibha Sharma [1], Jihong Xu [2] and Vinay K. Puduvalli [3*]

Fig 4 comparison of programmed cell death in NHA vs GSC262 and GSC811 (Color images)

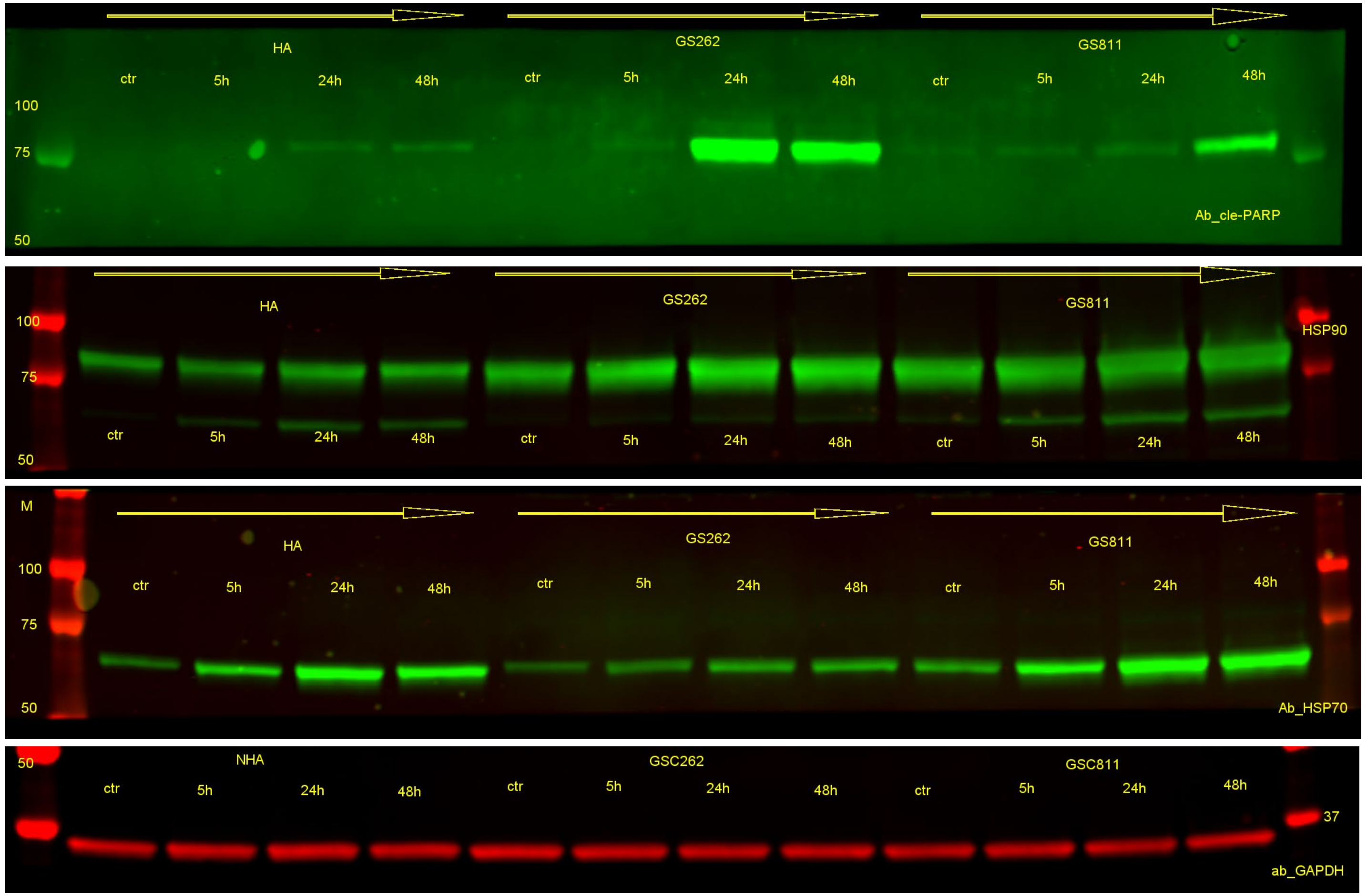

Fig 5 (a)PU-H71 dose response (color images)

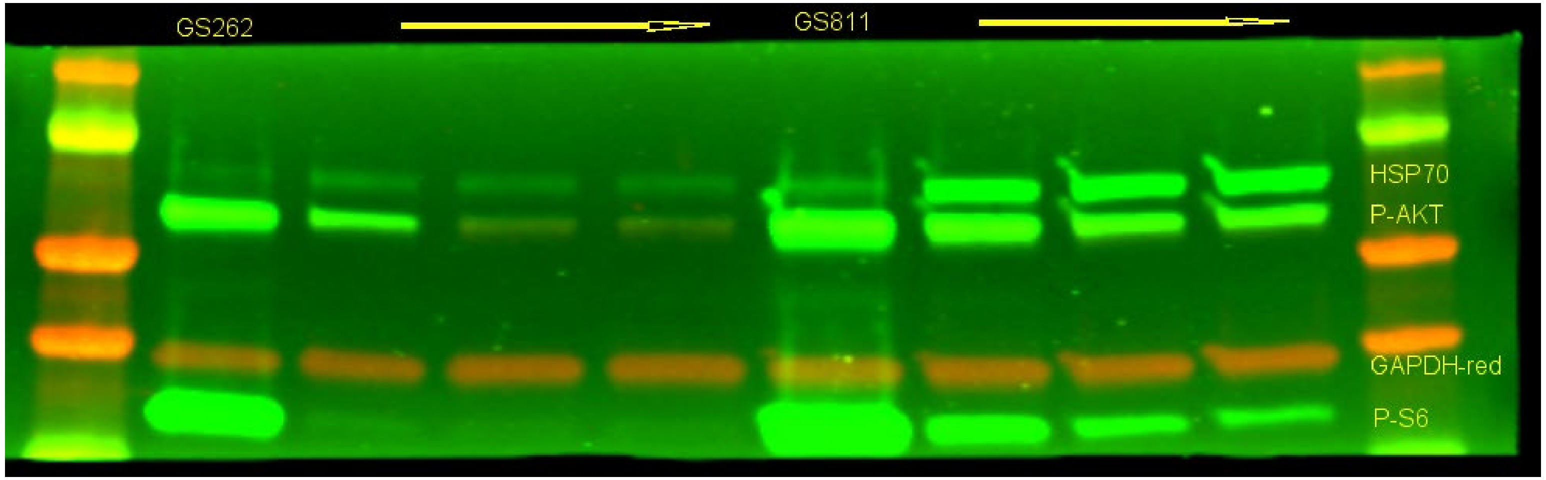

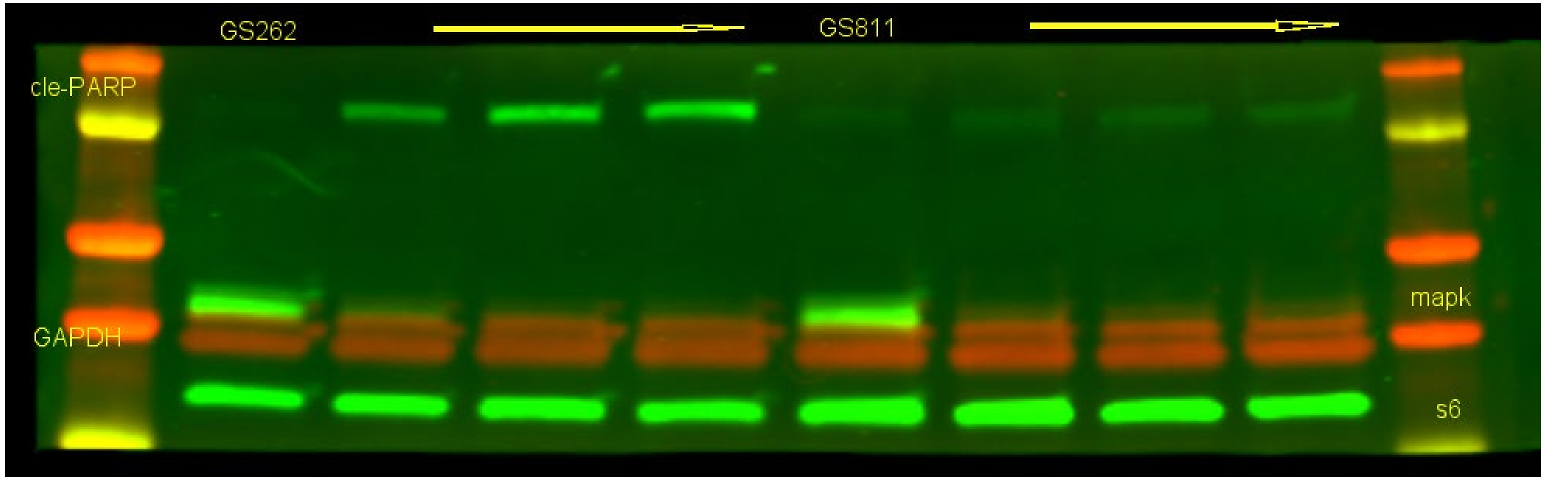

Fig 5 (b) PU-H71_Time Response (color images)

Blot-1

Blot-2

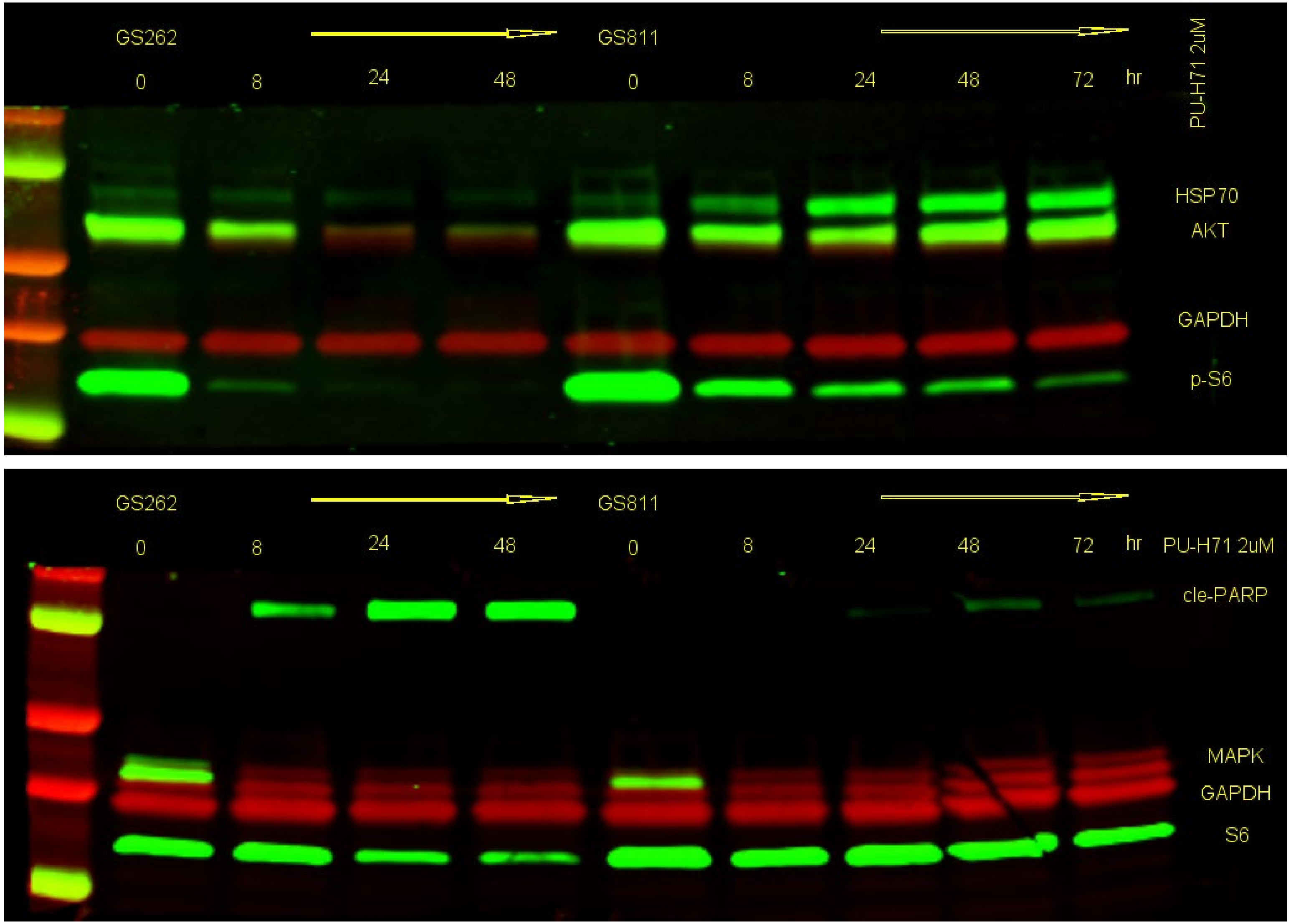

Supplement: Supplementary file 1 [file cancers-16-03934-s001.zip › cancers-3277282-supplementary.pdf]
